# Using Alternative Sources of Energy for Decarbonization: A Piece of Cake, but How to Cook This Cake?

**DOI:** 10.3390/ijerph192316286

**Published:** 2022-12-05

**Authors:** Dmitry V. Boguslavsky, Konstantin S. Sharov, Natalia P. Sharova

**Affiliations:** Koltzov Institute of Developmental Biology, Russian Academy of Sciences, 26 Vavilov Street, 119334 Moscow, Russia

**Keywords:** alternative energy, spaceship Earth paradigm, decarbonization

## Abstract

Few analytical or research works claim that the negative impact of improper use of ASEs may be comparable with that of hydrocarbons and sometimes even greater. It has become a common view that “green” energy (ASE) is clean, safe and environmentally friendly (eco-friendly) in contrast with “black” energy (hydrocarbons). We analyzed 144 works on systemic and/or comparative research of the modern and prospective ASE: biofuels, hydrogen, hydropower, nuclear power, wind power, solar power, geothermal power, oceanic thermal power, tidal power, wind wave power and nuclear fusion power. We performed our analysis within the Spaceship Earth paradigm. We conclude that there is no perfect ASE that is always eco-friendly. All ASEs may be dangerous to the planet considered as a closed and isolated unit (“spaceship”) if they are used in an inconsistent manner. This is not in the least a reason to deny them as prospective sources of energy. Using all ASEs in different proportions in various regions of the planet, where their harm to the planet and humanity can be minimized and, on the contrary, their efficiency maximized, would give humanity the opportunity to decarbonize the Earth, and make the energy transition in the most effective way.

## 1. Introduction

### 1.1. The Hydrocarbon Civilization

Since its very beginning, humanity has used hydrocarbons as its main energy resource [1,2,3]. An Old Testament nomad and a nineteenth-century worker both relied upon timber and charcoal or fossil coal to obtain energy through combustion [4]. The industrial revolution has catalyzed a fast transition to the omnipresence of hydrocarbons as primary sources of energy in almost every human activity, from industry to travel. Our modern civilization may be deservedly named the “Hydrocarbon Civilization”, as Vaclav Smil believes [5,6,7,8]. 

In the 1970s, H. E. Goeller and Alvin M. Weinberg from the Oak Ridge Institute for Energy Analysis published a number of papers in which they introduced the novel term “demandite” [9,10]. This “demandite” was described as an instrument—a non-existent, yet useful, “artificial mineral” whose composition demonstrates humans’ demand for various natural resources. Its composition for the worldwide estimations in 1968 was (CH_1.71_)_0.67_ · (SiO_2_)_0.21_ · (CaCO_3_)_0.08_ · Fe_0.0145_ · N_0.0068_ · O_0.0045_ · Na_0.0045_ · Cl_0.0045_ · S_0.0023_ · P_0.0007_ · K_0.0007_ · Al_0.0007_ · Cu_0.0004_ · Zn_0.0004_ · Pb_0.0004_ ·Mg_0.0004_ · (Mn + Ba + Cr + F + Ti + Ni + Co + Ar + Ne + He + Xe + Kr + Sn + Bi + As + Sb + B + Br + I + Zr + Au + Ag + platinum metals + rare earths + transuranium elements)_0.0008 in total_ [9]. In 2018, Wolfgang Sassin et al. recalculated the demandite formula for 2016–2018 and derived (CH_1.94_)_2.98_ (the demand for hydrocarbons being five times greater than in 1968), while the requirements for other elements were only 1.5 to 2.5 times higher than in 1968 [4]. The substantial increase in (CH*_x_*)*_y_* proportion can be explained by (1) the rise of the world population and (2) the heavier reliance on using hydrocarbons today [4,11,12]. 

This means that now we burn four to five times more fossil fuels than we did half a century ago, while the world population has only doubled. Surely, a part of the (CH*_x_*)*_y_* growth can be accounted for by the rise in hydrocarbon consumption unrelated to energy (e.g., using plastic materials, wearing synthetic clothes, or consuming engine oils). In any case, though, the contemporary increase in the amount of hydrocarbons being burnt is exceptional. 

From the demandite formula, we can see that the demand of humanity for hydrocarbons is much higher than for any other element or substance found on Earth, and it continues to grow rapidly. From 1968 to now, the product *x · y* in (CH*_x_*)*_y_* grew faster than the indices for other elements (groups of elements/functional groups/substances/compounds) in the demandite formula [4].

### 1.2. Carbonization of the Earth

**Figure 1:**
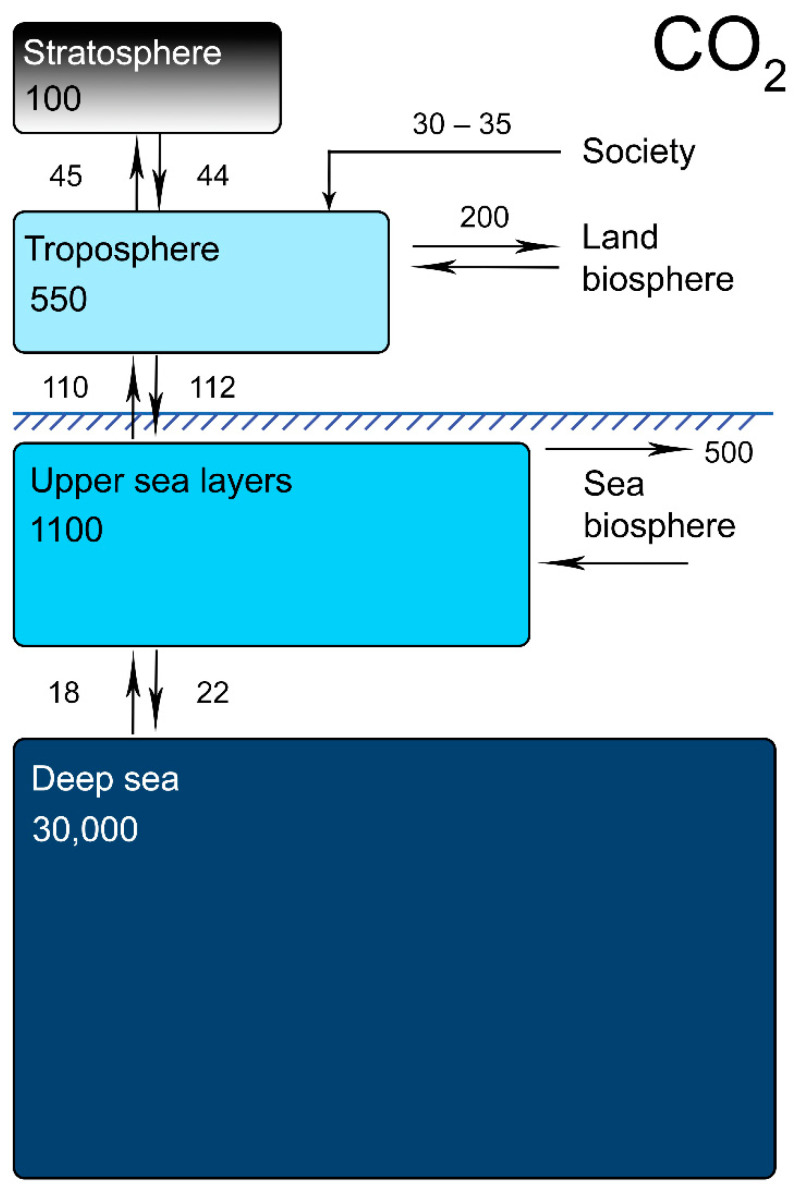
The largest natural reservoirs of carbon dioxide (billion tons) and carbon dioxide fluxes (billion tons per annum). Based on the data provided in [18,19,20,21].

As a result of our Hydrocarbon Civilization-related activities, around 3 trillion tons of CO_2_ have been emitted by humans since the first steam engines were used in the late eighteenth century [13,14,15,16,17]. The appropriate standard for comparison may be the quantity of natural CO_2_ contained in different parts of the planet and the annual CO_2_ flows between these parts [18,19,20,21]. Figure 1 summarizes this information. 

During the last five years, the average annual carbon dioxide emissions have been 30–38 billion metric tons [22,23,24]. As we can see, the current annual figure of anthropogenic carbonization is comparable with the natural flows of this gas between the troposphere and stratosphere. It significantly exceeds the ocean–lithospheric flux, and it is only three times less than the annual natural gas flux between the troposphere and the upper ocean layer. 

**Figure 2:**
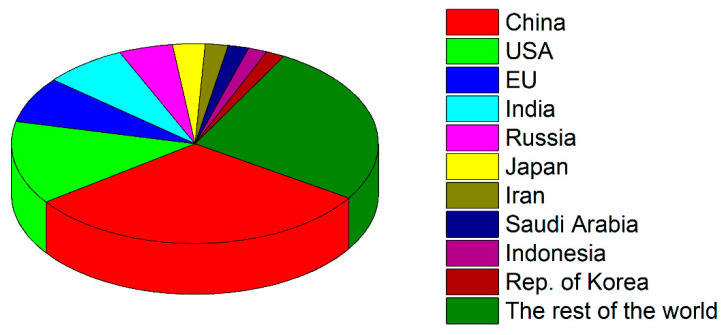
The proportion of different countries/political blocks in CO_2_ emissions in 2021, in descending order. The total amount was estimated as 37.124 billion tons of CO_2_ [25].

Not all countries contribute to the carbonization of the planet equally. Figure 2 demonstrates the proportions of different countries/political blocks in terms of CO_2_ emissions [25]. We see that the top ten carbon dioxide emitters contribute around three-quarters of all carbonization on Earth. China barely accounts for around one-third of all anthropogenic CO_2_.

The greenhouse effect of carbonization leads to a slow yet inexorable rise in the average temperature of the planet [26,27,28,29,30,31,32]. We must emphasize that, nowadays, we are observing not merely a rise in average temperature but an *accelerated* rise [29,30]. With approximately 15–20 billion humans expected by 2100 [4,31,32] and the ever-growing appetite of humanity for energy, the greenhouse effect will speed up. 

Many scientists, business entities, social activists, governments and policy makers agree that there is a need for a so-called “energy transition” from fossil fuels to alternative sources of energy (ASE) to escape the adverse effects of carbonization [33,34,35,36]. 

Environmentalist academia has developed different scenarios of the greenhouse effect and its curbing (e.g., +1.5 degrees, +2.0 degrees above pre-industrial levels) [31,32,33,34,35,36,37]. However, the Earth absorbs excess carbon dioxide slowly, while humans emit CO_2_ quickly. This is the main reason why even an ideal scenario with zero emissions in most states by 2100 will not automatically lead to the attaining of pre-industrial temperatures. There is always an inertial interval. In the case of the achievement of totally non-carbon energy production by 2100, pre-industrial temperatures may be reached no sooner than 2150–2200 [36,37]. Discussing such distant times is sheer speculation.

### 1.3. The Spaceship Earth Paradigm and the Alternative Energetics

In the 1960s, a new environmental paradigm emerged, according to which our planet is considered a closed “spaceship”. Barbara Ward [38], Buckminster Fuller [39], and Kenneth E. Boulding [40] equally contributed to the creation of this scientific worldview, though the very term dates back to the late nineteenth century [37].

According to this paradigm, the Earth is a virtual “spaceship”, from which we cannot eject any substances. Nor can we easily dispose of the excess energy produced by this spaceship. Therefore, the spaceship is closed and isolated from the inside in terms of physics. The spaceship is closed but is not isolated from the outside, as we may utilize energy from space. A key consideration of the Spaceship Earth paradigm is that the planet is a single interconnected unit.

In our review, we analyze ASE within the Spaceship Earth view. There is no lack of original articles, monographs and reviews devoted to ASE that have been published during the last four decades. In fact, they already amount to thousands [41,42,43]. However, very few allow a systemic view that would permit one to compare different ASEs, taking into account supply chains and energy flows on the planetary scale. We are certain that the Spaceship Earth paradigm provides flexible instruments for such an analysis.

The Spaceship Earth paradigm helps us to adopt the global perspective on using a certain ASE or a group of ASEs. A definition of the Spaceship Earth approach to ASE may be as follows: *The set of all preliminary steps, operation cycle and consequences of using an ASE*. 

The major areas of focus here are: (1) the production of the energy carrier (substance) (if any); (2) the production of facilities at which the ASE is generated; (3) the installation of facilities and infrastructure; (4) the transportation of the energy carrier (substance) (if any); (5) the operation of facilities and infrastructure; (6) storing and transporting the energy, if no tangible substance is present in the ASE. An example of a tangible carrier is hydrogen, as it is a source of energy and its carrier simultaneously. An example of an ASE without a tangible substance carrier is hydropower, solar power, or wind power. In the latter case, within the Spaceship Earth paradigm, significant attention has to be paid to the battery problem (relation between the source of energy and carrier of energy), which we shall discuss after analyzing the main ASEs.

The reader may be a little surprised by the title of our review. In their recent notable paper “Is the renewables transformation a piece of cake or a pie in the sky?”, Caroline Zimm et al. provided and discussed four arguments in favor of alternative energy transformation (in their words, “why renewables transformation could be a piece of cake and not a pie in the sky”) [44]. Agreeing with the major statements of the authors, we argue that preparing this “piece of cake” would be an exceptionally difficult task on the scale of the whole world, especially within the time limits 2025–2030, during which the main policies of the energy transition to ASE have to be set up.

## 2. Materials and Methods

This narrative review was composed according to the SANRA guidelines (Scale for the Quality Assessment of Narrative Reviews) [45]. 

To obtain the necessary literature sources, we screened the GeoRef, Science Direct and PubMed databases for the original articles, reviews and books published from 1980 to 2022 in English. Several relevant works beyond this date range or outside of the English language requirement were taken arbitrarily. 

The automatic search was performed in accordance with the following key words: alternative energy; hydrocarbons; renewable energies; biofuel; biogas; nuclear power; thermonuclear power; nuclear fusion; hydropower; wind power; solar power; geothermal power; sea tidal power; wave power; ocean thermal power; OTEC; hydrogen. The only items that were included in our review had been identified by at least four key words simultaneously. From the resulting list of 1348 works, we chose only those works that discussed different ASEs in a comparative or systemic manner. The inclusion procedure was carried out manually by two reviewers (D.B. and N.S.), independently of each other. The third reviewer (K.S.) helped to resolve issues of inclusion through discussions.

We added a number of technical references to the resulting list of 144 works. 177 works in total were included in the review. Data extraction was performed on this set using our proprietary manual for the collection of information relevant to the purpose of our review. 

## 3. Conditionally Renewable Alternative Sources of Energy

### 3.1. Biofuels

Biofuels may be the oldest ASE, as they date back to the ancient world. In modern times, humanity is attempting to reconsider them as prospective future fuels. The rationale behind their use is simple: we take biomass from the biosphere or atmosphere. 

Biofuels are commonly considered promising fuels by a number of modern authors [1,5,14,17,21]. They are conditionally renewable, as they may be exhausted due to the large-scale cutting down of woods (Supplemental File). In this chapter, we focus on a detailed discussion of their pros and cons.

#### 3.1.1. Solid Biofuels

This section concerns solidified biomass. Lumber, turf and charcoal were the three primary energy resources for many thousands of years, up to the beginning of the steam engine era [45,46,47,48]. Since the eighteenth century, in most societies, the use of raw lumber for industrial purposes has been exceeded by anthracite, bituminous coal and brown coal [49,50]. 

Today, in the Global South, timber is also rarely employed, even for personal use. Instead, solid biofuels have become popular. These are normally manufactured as pellets from biowaste or as the side-products of the food/sewing/lumbering industries, e.g., sawdust, straw, shavings, lumber chips, fabric scraps, olive bones, tree rind, nut shells or sunflower seed shells [51,52]. Manure, as a side product of livestock farming, is also an important resource for preparing solid biofuel tablets and pellets [53,54]. 

The attractiveness of solid biofuels consists in (1) the simplicity of their production, (2) their ability to be included in a closed resource cycle (within the borders of a self-sustained household), (3) the broad availability of ingredients, (4) the absence of a special storage regime, (5) their very long service life time and (6) their inexpensiveness [55,56,57,58,59,60]. 

A key solid biofuel is biohexamine (hexamethylenetetramine) fuel, which is commonly distributed in tablets. As a rule, hexamine, whether bio-based or artificial (industrial), is produced from two components, formaldehyde and ammonia, via depositing in crystalline form in a reactor [61]: 
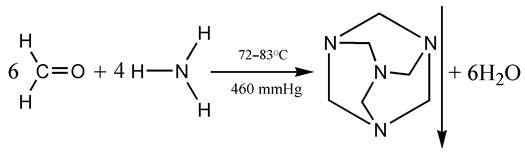
(1)

Bioformaldehyde can be obtained as a “natural” product of catalytic biomethanol oxidation and dehydration:
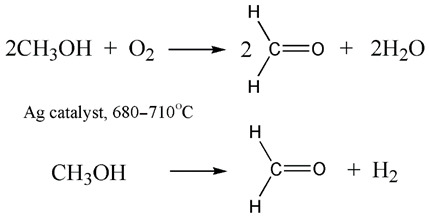
(2)
whereby biomethanol is derived via liquid biofuel synthesis from biomass using biosyngas [62,63] (see below).

Ammonia, the second component of hexamine synthesis, results from a catalytic reaction of hydrogen with nitrogen, gases that may be derived from water hydrolysis and from the air [64] anywhere on Earth. The power capacity of biohexamine is significantly higher than that of common solid biofuel, and it gives stable smokeless flames. This fuel is perfect for water heating systems in domestic households and public places, especially in rural areas. However, the production of biohexamine, despite being energy-efficient, is much more complicated, time-consuming and expensive than that of common solid biofuels [65].

As of late, the main applications of solid biofuels are domestic and small business heating systems, as well as agriculture. However, prospective smart villages are being designed to include small power plants with liquid energy carriers and turbines. They are deemed to work on solid biofuels and supply these self-sustained settlements with electricity [55]. 

#### 3.1.2. Liquid Biofuels

Solid biofuels are projected to be widely used in the future, whereas liquid biofuels are currently produced in comparatively substantial amounts [66]. In addition to this, solid and liquid biofuels have different applications. The former is generally used for heating and the latter for transport vehicles [67]. 

Liquid biofuels, for the most part, include bioethanol, biodiesel, biobutanol and bioethers [68]. All of these substances can be produced from green land biomass and algae [69]. The International Energy Agency (IEA) considers liquid biofuels as the most promising alternative for use in transport, including aviation, in the decades to come [70]. Today, biofuels constitute merely 3% of the world’s vehicle fuels, but the IEA has set a target of 25–30% by 2050 [71]. Europe, the UK, the USA and Latin America account for around 85% of the current worldwide production and consumption of biofuels [72].

Bioethanol is largely obtained from corn, tapioca (USA), freshwater and marine algae, cassava (the Caribbean basin), sweet potatoes, sugar cane, sorghum, milo (Latin America), crops, millet, sugar beet (Europe, Russia, the UK), or rice (China, Southeast Asia) fermentation, and can be mixed with petrol in any proportion [73]. 

Biodiesel is an oil-like substance that is mainly derived from plant oils (crude palm, soybean, rapeseed, sunflower, flax, mustard, or hemp oils) in a catalytic transesterification reaction with methanol [74]. Some biodiesels are produced using hydrocracked animal fats or used cooking oil [75,76].

Biobutanol (regardless of its isomers) is a second-generation liquid biofuel. Its energy capacity is larger than that of bioethanol [77]. Therefore, its use in internal combustion engines may be preferable over bioethanol, whether as a fuel itself or an addition to petrol. It is produced as a “natural” fuel via the fermentation of potatoes, sugar cane, sugar beet, corn, cassava, sorghum, wheat and cellulose, with the use of the bacterium *Clostridia acetobutilicum* [78]. Applying *Escherichia coli* as a fermentation agent is much less effective in terms of the yield of biobutanol [79]. Another advantage over bioethanol is the ability of Clostridia acetobutilicum to transform biomass that contains polymeric cellulose [80]. 

Bioethers are frequently synthesized from the waste glycerol obtained during biodiesel production [81]. Their synthesis is very cost-effective, but now they are chiefly used as octane number stabilizers in petrol and very rarely as fuels in their own right [82]. They are highly flammable, require a special storage regime and have a relatively low energy density. Consequently, they can be compared to the first three liquid biofuels in our list, in terms of neither energy effectiveness, nor cost effectiveness [83].

#### 3.1.3. Gaseous Biofuels

This group consists of biogas and biosyngas [84,85].

Biogas is a mixture of natural gas, carbonic acid gas and hydrogen sulfide. It is derived from rotting farm waste, sewage waters, and everyday domestic waste in anaerobic conditions using anaerobic bacteria [86]. Biomethane, which results from the purification of biogas, can be utilized in any area where natural gas is applied.

Biosyngas, or a mixture of carbon monoxide, hydrogen and light hydrocarbons, is derived during biomass pyrolysis and partial oxygenation [87]. The artificial analog of biosyngas is obtained via the well-known reaction of the vapor conversion of natural gas [87]. Biosyngas have a variety of industrial applications, just like their artificial equivalent [88]. For instance, biosyngas may be used for the production of biomethanol, a valuable liquid biofuel: 
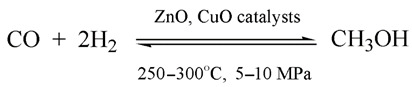
(3)

Biomethanol can be further utilized for the dilution of petrol in internal combustion engines.

The USA and continental Europe are the contemporary world leaders in producing and consuming gaseous biofuels [89]. 

#### 3.1.4. Summary for Biofuels

Biofuels are popular substitutes for fossil fuels, and their popularity is expected to increase in the next years. As they are conditional renewables, they address the problem of fossil fuels’ exhaustiveness. Nonetheless, within the Spaceship Earth paradigm, they are not so attractive. Here, we address why.

The prefix “bio-“ applied to this group of fuels is rather confusing. If one wishes to refer to their biological origin, it makes sense—though fossil anthracite, similar to pellets of manure, is also of biological origin since it is basically a carbonized and humified product of ancient vegetation found within the Earth. However, in political and media discourse, this prefix is often taken as a synonym of “biosphere-friendly”, “environmentally friendly”, “green”, or “suitable for sustainable development” [90]. This is not likely to be justifiable.

First, biofuels are environmentally damaging CO_2_ sources, just like fossil fuels. In spite of their production of lesser amounts of aromatic (e.g., phenol-formaldehyde) tars, carbon soot and long-chain organic pollutants than gasoil or fuel oil during their combustion, we ought not to forget that *they are hydrocarbons*. Their wide use would also contribute to the carbonization of the planet, regardless of whether or not their use may be economically justifiable in certain regions of the Earth. Therefore, upon burning, they transform into carbon dioxide, water vapor and an admixture of heavier hydrocarbon pollutants, including sulfurated ones. To believe that a fuel obtained from the biosphere, and thus a “bio” product, would be more “biosphere-friendly” in its utilization than a fuel excavated from the subsoil, is naive. 

Second, the broad transition to energy-effective liquid biofuels, e.g., biomethanol, is still expensive, at least with today’s technologies. Producing the artificial analogs of biomethanol or biobutanol is several times (sometimes more than a dozen times) cheaper, and the environmental result of combusting bioethanol or biobutanol is the same as that of combusting industrial methanol and butanol. Biogas and biosyngas are relatively cheap in small-scale production (cheaper than their industrial analogs), but their synthesis becomes economically ineffective at large scales due to the extra expenses related to the infrastructure of anaerobic tanks, pyrolysis areas, warehouses, and transportation, as well as the expensive chemical reagents and gas separation equipment [91]. 

Finally, the future mass reliance on some biofuels may lead to enormous damage to the biosphere. For instance, producing biodiesel requires large agricultural areas for rapeseed production in Europe, sugar beets in the UK and corn in the USA. The increase in world population, with the related growth of energy demand, would require a massive expansion of farming land for biodiesel production. This may be achieved only by deforestation and shrinking other reservoirs of nature for the sake of augmenting agricultural plantations. Likewise, the massive cultivation of sea algae in coastal areas to produce bioethanol would mean interfering in or even destroying natural marine ecosystems and consequently perturbing the biota of the world’s oceans. Using biofuels everywhere on the planet will lead to disaster. The Earth cannot provide sufficient amounts of lumber, algae and cultivation grounds for the energy requirements of eight billion people, let alone the fifteen or twenty billion that may be expected by 2100.

These ASEs may be temporarily important—and sometimes cost-effective—additions to fossil fuels but will not meet the final goal of global energy transition of the twenty-first century. 

### 3.2. Nuclear Energy

Nuclear fission in nuclear power plants is a non-hydrocarbon-reliant way of obtaining energy. However, due to the potential dangers of nuclear decay reactions to the environment, today a number of countries, such as Germany, have adopted prohibition policies and stopped most or all of their nuclear programs [92]. In continental Europe, Belgium, Italy, Spain, Sweden and Switzerland forbade the construction of new nuclear plants [93]. In November 2021, Austria, Denmark, Germany, Luxembourg and Portugal voted against the European Commission’s intention to regard nuclear power as a “green” energy resource [94]. 

At the beginning of the third decade of the twenty-first century, the proportion of nuclear energy within the world’s energy infrastructure is about 10%, or in absolute value, around 2500 Terawatt–hours (TWh) per annum [95]. One-quarter of the EU’s total electricity is produced today in nuclear plants [95]. Before the Fukushima accident in 2011, the worldwide proportion of nuclear energy use was expected to grow to at least 25% by 2050 [96]. However, now, its role will probably gradually decrease, both in Europe and in the rest of the world [97,98]. 

What may be the main objections against the broad construction of nuclear plants?

The first consideration is that, independently of the nuclear reactor type (thermal-neutron or fast-neutron), fuel or construction features, the radioactive decay of the actinoides that are used as reactor fuel (uranium or transuranic elements) produces a great deal of nuclear waste that cannot be effectively utilized [99]. The products of the bombardment of ^235^U with neutrons include more than three hundred radioactive isotopes of thirty-five consecutive elements from the Mendeleev periodical table, starting from bromine and reaching up to gadolinium [100,101]. These radioactive elements undergo *β*-decay, with the radioactive elements requiring storage in the lithosphere [102]. Radioactive heavy water is also a waste product of nuclear plants [103]. Radioactive waste (water or solid waste) must be earthed [104]. As a result, the radioactive pollution of the Earth increases. The danger of these radioactive wastes to the planet is substantial, especially if the part of nuclear energy is increased in the future.

The second objection is the vulnerability of nuclear power plants to human errors and natural disasters. The Windscale Fire, Three Mile Island, Chernobyl and Fukushima are, to date, the most catastrophic events that have led to massive radioactive pollution [105]. Despite all nuclear plants boasting multiple-level safety systems, the aforesaid accidents have demonstrated that no protection system is able to prevent the emitting of radioactive dust to the troposphere, hydrosphere and lithosphere as a result of the most severe accidents. In the cases mentioned above, the resulting radioactive contamination was vast in area and substantial in amount, even with all liquidation measures properly taken [105]. As a result, large alienation zones were created, in which most of the biota were totally destroyed by the highly radioactive elements, which have short-term and middle-term half-decay times. The most dangerous radioactive pollutants in the damage zones were ^137^Cs, ^90^Sr, ^113^Cd, ^121^Sn (middle-term), ^131^I, ^89^Sr, ^95^Nb, ^91^Y, ^140^Ba and ^95^Zr (shorter-term half-life times) [105]. The latter group is extremely hazardous to humans and biota due to its excessive radioactivity.

The third limitation of nuclear energy is that it leads to energy inequality across the world. With the discrepancy in public policy in different countries regarding the prospects of nuclear power plants (even within a political block, e.g., in the EU), nuclear energy will inevitably create additional energy disparity around the world. Countries in the Global South will be disadvantaged, as almost all nuclear plants are built and run in the Global North. In our opinion, a power that increases inequality in the access of people to energy cannot be the power of the future. 

## 4. Renewable Alternative Sources of Energy

### 4.1. Hydrogen: Splendor and Misery of Colored Classification

For the last twenty years, hydrogen has been widely discussed as a possible future road transport fuel and less discussed as a heating fuel used in small power plants. A number of hydrogen-based engine construction patents have appeared during this time. 

As an industrial, large-scale heating, marine, aviation, or spacecraft fuel, hydrogen will scarcely be broadly usable until 2030 [106]. The reasons are the construction difficulties related to increasing the energy efficiency of catalytic hydrogen oxidation in large turbines and the safety measures intended to prevent the possible formation and explosion of H_2_–0_2_ mixtures during full H_2_ combustion in nozzles [106]. On 22 February 2022, the Airbus corporation announced the first hydrogen-fueled plane to be released by 2035 [107], but the validity of their announcement is not yet supported by any publicly disclosed scientific research. We have to remember that hydrogen is a dangerously explosive gas, and its mixture with oxygen in the proportion 2:1 can lead to spontaneous explosion. 

Upon its catalytic oxidation or combustion, hydrogen produces water, the most common and safest liquid on the planet. However, not all hydrogen is considered a prospective “eco-friendly” energy resource.

A color-related word system is used for hydrogen in political discourse, media and some environmental programs [108]. Hydrogen is marked in these cases as follows:

(a) **Grey**—H_2_ produced from or with the use of fossil fuels with carbon emissions to the troposphere.
  (1) The majority of industrial hydrogen (around ¾) is now obtained in the process of steam-reforming natural gas, pure methane or light alkanes such as propane–butane fractions [109]. Artificial syngas and carbon dioxide are obtained as the products of the following reactions:

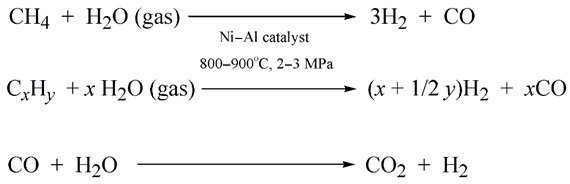
(4)
    Water vapor further oxidizes the carbon monoxide in the syngas to carbon dioxide (the third reaction in Equation (4)).
  (2) Hydrogen is also currently derived in industrial amounts from water vapor over petroleum coke. This is the oldest means of industrial hydrogen production, which dates back to the early nineteenth century [109]:

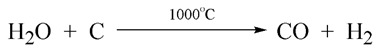
(5)
  (3) A small part (less than 10%) of hydrogen is obtained by the catalytic or temperature-based partial oxidation of natural gas [109]:

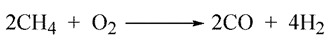
(6)
(b) **Blue**—Everything is the same as for the grey category, but carbon monoxide and carbon dioxide are stored in tanks.

Later, when CO is oxidized, CO_2_ is pumped into the Earth’s crust through lithospheric cracks. These processes are referred to as carbon capture and storage (CCS), carbon capture and utilization (CCU) and carbon capture, utilization and sequestration (CCUS) [110];

(c) **Yellow**—H_2_ is produced via the reaction of metals with acids.

This is a common laboratory-based process of hydrogen synthesis [109]. In this example, copper reacts with a water solution of sulfuric acid and produces gaseous hydrogen:
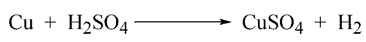
(7)

(d) **Green**—H_2_ is derived from the electrolysis of water:

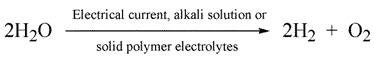
(8)

(e) **Turquoise**—H_2_ is obtained via the reaction of natural gas pyrolysis [111]:

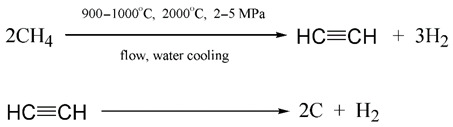
(9)

In the pyrolysis process, other hydrocarbon products are usually also formed in addition to acetylene, such as ethylene, propylene, butadiene (diacetylene), vinylacetylene and butane [111]. Thus, producing turquoise hydrogen is as environmentally unsafe as producing grey hydrogen, as it increases the carbonization of the planet (soot and unsaturated hydrocarbons).

The acetylene formed in the first process of Equation (9) is unstable at temperatures over 1000 °C and may easily be decomposed to soot and additional hydrogen (the second process in Equation (9)) [111]. As such, if the production of acetylene is not planned, pyrolysis may be carried out up to the maximum amount of hydrogen;

(f) **Pink**—H_2_ is derived from the high-temperature (800–1000 °C) electrolysis of water, using the electricity and heat generated in nuclear power plants.

A ceramic material made of costly ZrO_2_ + Y_2_O_3_ serves as the electrolyte for transferring the O_2_^−^ radical—this ensures the electrical current [112]. Today, about 0.1% of all hydrogen is obtained using this “pink” method [112].

The “pink way” is a specific approach to hydrogen generation, whose cost efficiency is relatively low. The exclusive use of heat energy derived from other sources and cheap electricity, e.g., those derived from nuclear plants, may help to reduce the cost of “pink hydrogen” production.

One can be easily dazzled by this hydrogen-related rainbow. 

These colors are used to reflect the degree of eco-friendliness of hydrogen obtained in different ways [108]. As one can easily infer, “green hydrogen” is much more preferred in media, as well as by a number of politicians and business representatives, as a prospective eco-friendly energy source—sometimes to the extent that only “green hydrogen” is designated as a possible green fuel for hydrogen-powered engines [113,114]. However, we have to ask, to what extent is producing “green hydrogen” environmentally safe and economically reasonable?

Frankly speaking, any water electrolysis approach that yields “green hydrogen” is economically unviable in the current conditions [115]. Common (water solution) electrolysis is very expensive (the production cost is around USD 10 per kg of 96–98% gaseous H_2_), while solid polymer membrane electrolysis is immensely expensive (around USD 27–32 per kg of 99.999% gaseous H_2_) [116]. In comparison, the steam conversion of natural gas produces “grey hydrogen” with a production cost of 10–12 cents per kg of 95–98% gaseous H_2_, 50–60 cents per kg of 99.9% gaseous H_2_, and about USD 2 per kg of 99.99% gaseous H_2_ [116]. That is, the steam reforming of natural gas (“grey hydrogen”) is one hundred times cheaper than common water electrolysis (“green hydrogen”) and three hundred times cheaper than solid polymer membrane water electrolysis (producing even greener hydrogen). The degree of purity of hydrogen is essentially irrelevant to its use as a vehicle fuel, for which 95% purity is quite sufficient [117]. 

Even “pink hydrogen”, obtained via high-temperature electrolysis in nuclear plants, is much cheaper than “green hydrogen”, viz. USD 2.4–4.0 per kg of 94–98% gaseous H_2_ [118].

In addition to its expensiveness, common (water) electrolysis is environmentally polluting. Indeed, the electrolysis of pure water is impossible and would be ultimately ineffective, as water is an electrical insulator (distilled water has a specific resistance of around 20–80 Kiloohm–meter (kOhm · m) [119]. Therefore, to ensure the electrical conductivity of water, concentrated electrolytic reagents are required with lower cationic potential than the hydroxonium ion H_3_0^+^ emitted on the anodes during electrolysis [120]:
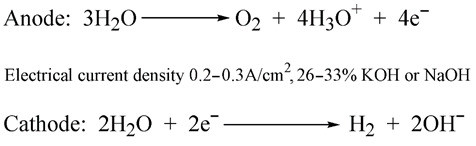
(10)

In practice, 26–33% alkali solutions of caustic soda or caustic potash are used (Equation (10)) [120]. These are hazardous to all biota, and they have to be utilized after several dozen cycles. The utilization process includes acids that are also environmental pollutants, especially when used in industrial amounts.

Solid polymer membrane water electrolysis does not require eco-dangerous saturated alkalis. Nor does it require large and clumsy electrolysis baths/tubes, pumps, adapters, valves and gas cylinders. Therefore, it could potentially be used not only in stationary hydrogen-producing plants but also in hydrogen-fueled vehicles [121]. However, as we saw above, the technique is extremely expensive and cannot be applied in mass hydrogen-fuelled transport production—at least, not yet.

To sum up, the current problems of using hydrogen fuel become obvious when we adopt the viewpoint of the Spaceship Earth paradigm. We must not merely consider the safety and eco-friendliness of a hydrogen-fuelled car or a small hydrogen turbine. This is the Spaceship Earth dilemma—the car itself will be eco-friendly, as its engine will emit only water vapor. However, producing and transporting hydrogen that will fuel the cars will not in the least be eco-friendly. The following problems should be considered:

(a) Nowadays, only “grey hydrogen” production is economically reasonable. “Green hydrogen” is still impossible to obtain in massive amounts due to its high production costs;
(b) However, “grey hydrogen” is not at all environmentally friendly, as the very same hydrocarbons that lead to the greenhouse effect are used here as the reagents for hydrogen production;
(c) Not all “green hydrogen” is truly “green” either. The “green hydrogen” obtained via water solution electrolysis entails the alkalization or acidification of the environment (with the dangerous contamination of soil and fresh water reservoirs, causing biota extinction). Using such “not-so-green” hydrogen in eco-friendly cars would also entail the operation of eco-hostile hydrogen-producing plants;
(d) “Blue hydrogen” is also not an eco-safe option, as the huge-scale pumping of CO_2_ into lithospheric splits would increase the total carbonization of the Earth. In time, the carbon dioxide pumped in such a way will be released into the troposphere due to natural gas exchange processes (Figure 1);
(e) Let us assume a case in which humanity has successfully switched all its transport to operating on “green hydrogen”, having somehow dampened its cost inefficiency—this would still not solve the problem of the global energy demand for transport if we adopt the Spaceship Earth viewpoint. The global use of “green hydrogen” would, in fact, involve redistributing energy from one place to another.

The most important thing is that the Spaceship Earth view problematizes the very notion of “green” hydrogen. Theoretically, water electrolysis requires the same amount of energy to produce hydrogen as is being produced by hydrogen catalytic oxidation or full combustion—this is an unavoidable result of the energy conservation law for reversible chemical reactions. 

In real life, this would mean that more energy is required for hydrogen production than is produced by its combustion. Currently, the energy consumption related to water electrolysis is 4.5–6.0 Kilowatt–hours per cubic meter (kWh/m^3^) of H_2_ when using water solution tanks (with an efficiency coefficient of around 60%) and 3.7–3.9 kWh/m^3^ of H_2_ when using solid polymer membrane electrolysis (efficiency coefficient of around 72–80%) [122]. However, the best and most effective modern hydrogen engines are capable of producing only 2.5–3 kWh from a single cubic meter of H_2_ (recalculated to atmospheric pressure and 50–90 °C, which conditions are customary in water electrolysis) [123]. 

As we see, a great deal of effective energy of hydrogen is lost because of imperfect electrolysis bath and engine construction. As such, universally using “green hydrogen” would gradually diminish the fresh water resources of the planet, virtually transforming hydrogen into a non-renewable.

Hydrogen is a promising fuel for local applications. It can be energy-effective and eco-friendly in a local area, where its use is condensed, on condition that the financial, energetic and pollution expenses related to its production, storage and transportation can be neglected in other places. The smaller the distance between the hydrogen production site and its application venue, the lower the additional spending required and the potential environmental hazards incurred. In addition to its explosiveness, the negative properties of hydrogen include its low density and high diffusion plasticity—at high pressures and elevated temperatures, hydrogen is able to diffuse through many metals [124]. For this reason, long hydrogen pipelines (such as those that are used today for natural gas transportation) are not an option, as the environmental impact of their construction and functioning is unpredictable.

The option of using hydrogen as a fuel quickly becomes dubious when considered on the scale of the whole world according to the Spaceship Earth concept if we take that at least ¾ of the world’s transport will be fueled in this way [4,38,125]. The universal repumping of energy and the transfer of environmental pollutants from one location to another using hydrogen as a carrier would result from the worldwide use of hydrogen. 

Consequently, in the coming years, we have to focus on the most promising hydrogen-related techniques:

(1) Reducing the cost of solid polymer membrane electrolysis systems;
(2) Increasing the safety and capacity of membrane-adsorption or metallic-adsorption hydrogen storage (e.g., multilayer rare-earth-based metallic “hydrogen accumulators” [126,127]);
(3) Augmenting the membrane electrolysis efficiency, as well as the efficiency of hydrogen-powered engines.

Achieving these goals would enable us to install easy-to-use electrolysis/accumulator systems on vehicles while eliminating the additional costs of hydrogen stationary production, transportation and intermediate storage. Enhancing the safety of hydrogen-fuelled engines might also permit their use in aviation and marine transportation. Hydrogen-powered “steam engines” will also be a practical option in railroad locomotives. 

All this being said, using hydrogen would rapidly become energy-ineffective if the proportion of hydrogen-fuelled transport rises to above 50–55% around the world. Hydrogen fuel is effective only when it is a means of obtaining energy and not when its global redistribution is required. This must be remembered, independently of what type of hydrogen production humanity will rely upon, be it crimson, maroon, mauve or azure.

### 4.2. Hydropower

Transforming the kinetic energy of river flows into electricity via turbines dates back to the 1880s [128]. Due to hydropower being a relatively old type of alternative energy, many researchers do not regard it as a true ASE [128]. Now, hydropower contributes around 15% of worldwide energy production, and the current leaders in net hydropower production are China, Canada, Brazil, the USA, and Russia [129].

Hydropower is a very cheap renewable ASE that requires little maintenance supervision [128,130]. The main cost of electricity produced by a hydroelectric power station before it breaks even is the cost of construction [128]. After the economic break-even point, the station produces even cheaper electricity [128]. Furthermore, many hydroelectric stations have high durability and can operate for many decades [128]. These are the main reasons why many countries now utilize their hydropower potential to a large extent. For example, Switzerland now uses nearly 90% of its total hydropower potential and has almost no space to enhance its proportion of use of hydropower electricity [131].

Notwithstanding its economic reasonability and maintenance simplicity, hydropower has a large negative environmental impact [130]:

(a) The building of large hydroelectric dams on plain rivers has led to the submerging of vast areas, which are now occupied by shallow water storage reservoirs. This severely damages the land biota and ecosystems of these areas.
(b) The overall rates of river flow decrease significantly. This leads to an increase in water temperature and the appearance of duckweed. Consequently, the distortion of river biota from the source to the estuary is a common effect and is not just seen in the reservoir.
(c) Migratory fish populations usually go extinct in rivers with hydropower dams.
(d) Mountainous hydroelectric stations often cause new lithospheric cracks, mud flows, the silting of the riverbed, shifting sands, earthquakes and avalanches.
(e) The uncontrolled dumping of water from water reservoirs, which usually lasts for 10–15 days, results in stress perturbances of ecosystems. This may cause the irreversible contraction of trophic chains, the shrinking of freshwater fauna populations, the death of river invertebrates, the disappearance of nesting areas of migratory birds, the insufficient humidification of flood soil, the decrease in freshwater algae/river plant biodiversity, and the diminishing of the flow of biomass to the sea.
(f) The most unexpected negative effect of hydropower stations is the fact that their water storage reservoirs may end up carbonizing the troposphere with natural gas that is synthesized during the anaerobic decay of flooded plants in shallow and warm waters. Methane causes the greenhouse effect to unfold even faster than carbon dioxide. Hydropower is an ASE that is expected to reduce global warming. Instead, it frequently contributes to it.

Hydropower is, by and large, the cheapest ASE. That is why many countries, especially states in the Global South, continue to invest large sums in the construction of new dams, despite hydropower being possibly the most environmentally dangerous ASE in wide use today. Nuclear power plant malfunctions that may lead to considerable radioactive pollution are rare. However, the biospheric destruction as a result of the construction of hydropower stations will be observed in 100% of cases.

As hydropower is so eco-hostile, we cannot consider it as a prospective renewable from the Spaceship Earth view. Hydropower is an instructive example of how an ASE being renewable does not guarantee its ecological safety [38]. Therefore, it would be highly advisable to diminish the proportion of hydropower being used and phase out hydropower stations gradually, along with restoration procedures that may ameliorate the damage done to the biosphere.

### 4.3. Wind Power

This energy source, along with hydropower, is usually referred to as “traditional”, since humans have been utilizing it since long before the industrial age [132]. The use of windmills in Europe dates back to the early Middle Ages, and sailing vessels have been dominating from the beginning of history up to the mid-nineteenth century [133]. 

Wind power resembles hydropower in that it has already been sufficiently tested for industrial purposes [134]. Many countries invest heavily in the future development of wind energy [134]. The part of wind energy already exceeds that of hydropower in a number of states. Denmark produces half of its electricity using wind generators, Ireland a third, Germany around a quarter, and Spain and Portugal around a fifth [135]. Nearly 760–770 Gigawatts (GW) of energy are currently obtained worldwide from wind [135]. 

From the Spaceship Earth perspective, the proportion of use of hydropower can scarcely be increased any further in many states. Increasing its use on a global scale will inevitably have a very hazardous environmental impact. What are the potential outcomes of the further development of wind power?

One positive is that wind generators reduce carbonization significantly. The Global Wind Energy Council has stated that wind power will help to reduce carbon dioxide emissions by 1.5 billion tons per annum from 2030 [135].

However, wind power is imperfect, considering its environmental impact. The negative effects of wind power on the troposphere, biosphere and society are as follows:

(a) Perpetual high levels of noise. One middle-size wind generator with a 250 Kilowatt (kW) power output creates around 50–80 decibel (dB) of noise up to 0.5–1 km away and more than 100 dB in areas near it. Larger mills are much noisier [136].
(b) It can generate infrasound that is especially hazardous to water and soil biota, as it swiftly spreads in dense media [137].
(c) Vast zones of human and biotic displacement are being established near wind power plants.
(d) The dispersing of natural wind currents may occur in the case of global reliance on wind power. This may even lead to changes in the climate in a given area. The climatic conditions may thus become drier and more continental [38,138].
(e) The rotation of the fans of turbines generates a substantial level of radio interference [139]. This necessitates building additional retranslating stations for TV, mobile networks and other communications around wind power plants, which may additionally perturb biota.

These effects are supplemented by the geographical considerations that prevent the equal distribution of wind generator plants around the world. Windmills are economically justifiable on sea shores only, where the wind blows with sufficient speed and consistency [135]. Therefore, only states with (1) large coastlines and (2) constant strong winds can utilize wind power effectively, e.g., countries in the North Sea or Mediterranean Sea regions. This creates the problem of storing and transporting electricity to other regions where wind generator plants are ineffective.

In addition to this, wind power plants cannot work on a 24/7 basis. They generate electricity unevenly on a daily, monthly and even yearly scale—essentially, they only work effectively when the wind blows strongly [134]. Consequently, wind-produced electricity has to be stored in batteries, and the periods of wind power plants’ inactivity ought to be compensated for by the use of other ASEs in the relevant territories.

### 4.4. Solar Power

Sun power has been used for cooking/warming up food, domestic heating households and agriculture for at least three thousand years [4]. For this reason, it may also be called “traditional”, just as hydropower and wind power are. Along with wind power, many governments have already set up programs for the transition to solar power [4].

The current proportion of use of solar energy roughly equals that of wind energy, viz. 760–770 GW and its contribution to global energy production is also growing [140]. 

In contrast to wind power, which is mainly utilized in large power plants, solar power can be used both in large energy stations and in domestic households and small-scale agriculture operations. This is possible because solar batteries of any size can be situated anywhere—mainly on the roofs of houses, storage houses and greenhouses [141]. Solar power is “calm”, and no undesirable noise is produced.

The benefits of solar energy include its usability in almost any condition as long as there is sunlight, independently of the temperature conditions of the area. In mountainous regions and highlands (e.g., in the Himalayas), solar power is sometimes the only possible source of energy [142]. It is one of the very few ASEs that can be effectively deployed in space [143]. It can also be used at the upper sea level to power sea agriculture or research equipment [144].

This notwithstanding, solar power has several significant limitations that prevent it from being used everywhere on Earth and increasing its use proportion. The most serious limitation is that it is only effective in territories with high mean annual brightness and low humidity (clouds should not cover the sun) [4,38]. In practice, this means that only land areas within 40° north to 40° south, and a limited number of polar/circumpolar regions (e.g., Antarctica, Greenland), are suitable for the construction of large solar power stations [4]. In addition to this, solar energy is not void of negative environmental impacts:

(a) Multilevel rotating solar power modules are only effective in large solar power plants that track the daily movement of the sun [145]. This technique involves large rotational glass reflectors and metallic infrastructure. The areas around industrial solar power plants become enormously hot. The condensed sunlight causes much of the biota to migrate from the areas surrounding sun power plants [38]. Therefore, even greater zones of social and biotic displacement are being created;
(b) In contrast to wind power, the operating of which causes the most harm, in solar power, preparing the solar elements is the most damaging to the environment.

Producing solar modules requires Si, As, Te, Ga, Cd, Se, In, P, and a number of other elements that are toxic either by themselves or when in used compounds with hydrogen and/or halogens, which are used in the production cycle [146]. Silicon is probably the least environmentally dangerous, but silicon-based solar elements have relatively low-efficiency coefficients [146]. Much more effective GaAs solar modules use AsH_3_ and PH_3_ and sometimes their halogenated variants [147]. These substances are extremely toxic to animals, including humans, even in trace amounts. Therefore, the worldwide large-scale production of solar batteries is very environmentally risky in relation to plant construction;

(c) Solar power plant equipment is complex. Automatic complex cleaning systems are necessary for removing atmospheric precipitation that may reduce the efficiency of solar energy conversion. The constant monitoring of solar panels is also a prerequisite for the normal maintenance of a solar power plant [145]. These factors increase the production costs of solar energy, which are substantially higher than the production costs of wind energy [140];
(d) An unobvious yet serious limitation related to the largest solar power plants (dozens to hundreds of square kilometers) is their ability to cause reductions in air temperature in surrounding areas. The more solar energy that is being removed from tropospheric regions above a given area (to be converted to electricity), the cooler the air in this area [38].

This will lead to water vapor condensation. Clouds and fog will inevitably appear, which will diminish the efficiency of the solar electrical plant. To restore the efficiency, either the overall power of the solar power plant will have to be diminished, which is unreasonable from an economic viewpoint [38], or chemical reagents for artificial precipitation will have to be dispersed in the troposphere above the plant, in order to stimulate precipitation (e.g., AgI aerosol) [148,149], which is unreasonable from the ecological perspective.

### 4.5. Geothermal Power, Ocean Thermal Power and Sea Wave Power

These ASEs are rather more exotic in comparison to those we have already considered in the review. Not much electricity is currently being obtained using these approaches (around 0.2% worldwide in total) [150].

The usual argument of those who advocate using geothermal power is the virtually limitless heat resources found in the Earth’s lithosphere, mantle and core [151]. Indeed, the overall power flux from the Earth’s subsoil to its surface is 45–50 TW, and this energy resource is sufficient to fulfill modern humanity’s energy demands [19]. This energy flow is spread very unevenly across the planet’s surface [151]. Building geothermal stations, be they petrothermal or hydrothermal (depending on the energy carrier), is only reasonable in places where the geothermal energy carriers are near the surface, i.e., in places of elevated volcanic and seismic activity [152].

However, we ought to understand that the eco-hostility of geothermal stations is so high that any possible future reliance on this ASE will by no means facilitate the protection of biodiversity and nature in general [152,153]. 

The environmental impact of a geothermal station, as compared to a solar or wind power plant of comparable energy output, is much higher [38]. The salinization of soil, the complete loss of biota (both fauna and flora) due to the dumping of hot mineralized water, earthquake provocation, new volcano and geyser formation and the tremendous noise produced by the escaping vapor together comprise a non-exhaustive list of the common effects of geothermal power stations [153]. 

Ocean thermal electrical conversion (OTEC) utilizes the temperature difference between well-heated upper sea levels and deeper waters that are much colder in powering heat engines [154]. This is still an emerging ASE. Despite the fact that the technology is nearly 140 years old, not many OTEC plants have been constructed and used to date [155]. The local application of either of the two types of OTEC (closed-cycle or open-cycle) may be effective only in equatorial and tropical zones of the world’s oceans, i.e., in places where the temperature difference between the water’s surface and deeper layers is maximal (around 25 °C) [155].

Theoretically, OTEC’s efficiency is limited by the efficiency of the Carnot’s cycle [156]. For the temperature difference of 25 °C in equatorial/tropical seas it will be

(11) $$k=\frac{\Delta T}{T_{heater}}\approx \frac{25K}{302K}=8.28\%$$

In real life, this is much lower due to heat losses in the infrastructure [156]. In a few OTEC stations built since the 1970s, the efficiency coefficient is around 2.5–3% [154]. Open-cycle OTEC, wherein the energy carrier is sea water, is regarded as a relatively environmentally safe ASE [154]. Closed-cycle OTEC stations normally use ammonia as a carrier, which is toxic to most species of water biota, including plankton, and there is a risk of emitting large amounts of this chemical into the upper sea level. With modern technologies, constructing and operating OTEC stations is very costly compared to the more traditional ASE.

Sea wave power may be utilized in (1) tidal power stations and (2) wind wave power stations. The former utilizes the kinetic energy of tidal forces, and the latter the kinetic or potential energy of sea waves. Both ASEs are currently very seldom used worldwide. There are several hundreds of tidal power plants around the world [157], but there are still no wind wave generators of industrial capacity, despite many projects having already been tested in the laboratory environment [158]. 

Tidal power stations are tolerably eco-friendly but very costly to build and maintain [159]. This factor is likely to have hindered their use up to now [160]. As regards the prospect of wind wave stations, no convincing calculations have been performed so far regarding their economic effectiveness. 

A major issue of OTEC and wave power plants is the transportation of electrical energy generated at sea, far from the coastline. This has to be delivered to the coast either by wires or in batteries on dry cargo ships. The former method is energy-ineffective without large alternative-current voltage-increasing substations, which must therefore be built on the sea bottom—this will increase the environmental hazardousness. The latter approach is environmentally risky a priori due to the possibility of water contamination in cases of cargo ship accidents.

### 4.6. Thermonuclear Power

Hundreds of types of nuclear fusion reactions are known to scientists [161]. In nature, they occur in stars [161]. However, only a few types of reactions are practicable under artificial conditions on the Earth, and no reaction has yet been developed for the industrial generation of energy [162]. 

In stars, the extremely high temperatures (several tens of million to one billion K) required for nuclear fusion, are common. On the Earth’s surface, there are no solid compounds that can endure temperatures of 10,000 K [163]. The most high-temperature-resisting solid substance identified thus far is a type of ceramic made of nonstoichiometric hafnium carbonitrides HfC_x_N_y_, which can endure temperatures of around 4500 K [164]. A possible approach to artificial nuclear fusion, which requires extremely high temperatures, is the creation of self-sustained rotational burning plasma, which will not contact any solid surface due to its being supported by a powerful magnetic field. This field will maintain the plasma in a circulating toroid away from the solid walls of the reactor [162].

However, this approach is only theoretical. In practice, the multiple experiments that have been performed in many countries since the early 1950s have given no positive results that can be replicated [162]. Today, the International Thermonuclear Experimental Reactor (ITER) in Saint-Paul-lès-Durance, France, is the most promising international project, which may finally provide the long-expected outcome [165]. However, the first repeatable experimental demonstrations have been postponed several times [165]. The results are now not expected until 2025, according to the agenda issued by the ITER directorate [166]. 

The burning of hydrogen ^1^H (the proton–proton cycle) is the most basic reaction that takes place in nuclear fusion—it is the most widespread and most energy-productive reaction in the universe [167]. Around 98% of all visible light in the universe is believed to be the result of the fusion reaction of hydrogen burning in stars [163]. 

However, the burning of hydrogen cannot be employed in artificial nuclear fusion. The full energy outcome of the proton–proton cycle is 26.7 MeV [167]. This is a high-energy output. This notwithstanding, some of the reactions of the proton–proton cycle have a very small nuclear section [167]. That is, in hydrogen fusion fuel, a negligibly small proportion of the particles will react with each other, leading to helium-4. To make hydrogen ^1^H reactors commercially profitable, they must be several thousand kilometers in diameter [167]. This is an extraordinary size, which would entail unbelievable construction, production and maintenance costs and huge negative environmental impacts. The burning of hydrogen is only energetically productive on the scale of stars and not in artificial conditions on the Earth.

Deuterium–tritium (17.6 MeV), deuterium–helium-3 (18.4 MeV), deuterium–deuterium (3.3–4.0 MeV), deuterium–lithium-6 (22.4 MeV), hydrogen–lithium-6 (3.0 MeV), helium-3–lithium-6 (16.9 MeV), helium-3–helium-3 (12.9 MeV), hydrogen–lithium-7 (17.2 MeV) and hydrogen–boron-11 (8.7 MeV) reactions are theoretically possible candidates for the operation of nuclear fusion industrial reactors [168]. 

For nuclear fusion to proceed at sufficient rates and with a sufficient proportion of particles (around 0.001% of all substances) to break the Coulomb barrier, these reactions will require working temperatures of around 100 million–10 billion K [168]. In artificial nuclear fusion reactors, sometimes, higher temperatures than are found in stars’ cores are needed for igniting the self-sustained plasma due to the Lawson requirement (the product *n_e_ · τ · T* of plasma density *n_e_*, the time of energy confinement *τ* and temperature *T*, must exceed the lowest limit level for the plasma reaction to being energetically profitable) [169]. 

Most of the work at the ITER is focused on preparing the conditions for the lowest-temperature deuterium–tritium process, which has a large section and is therefore easy to ignite, even at 100 million K [161]:
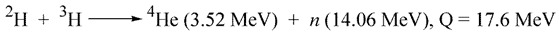
(12)

This reaction has a maximum section of 5 barn (5 × 10^−28^ m^2^) in resonance at the ^2^H energy of 0.108 MeV [161]. This is a very large value in comparison with the other promising fusion reactions (see above), which have sections of several hundredths of a barn. This makes the deuterium–tritium process the most suitable for practical use in prospective industrial thermonuclear power reactors.

A proper maintenance regime for a prospective thermonuclear power plant will ensure tolerable eco-friendliness. In case of an emergency, the radioactive emissions into the environment (consisting mainly of tritium) will be thousands of times lower than those produced by a nuclear plant [162].

The most persistent problem with prospective thermonuclear power plants is the serious high-energetic neutron radiation (Equation (12)), which will cause the materials normally used in the most secure modern nuclear power plants to become unusable in less than a year (the neutron radiation will be at least 100 times higher than that in today’s most powerful nuclear reactors) [170]. The short service lives of the equipment in thermonuclear power plants will increase the demand for construction materials, with inevitable delayed effects on the environment.

Most importantly, nuclear fusion exceeds all other ASEs in terms of efficiency coefficient and energy net value, but it is significantly inferior to other ASEs in terms of its astronomical research and development (R&D) costs [162]. One milligram of deuterium–tritium mixture can produce 337 Megajoules (MJ) of energy at 100 million K, which, as mentioned above, corresponds to 0.001% of the fuel mass [171,172,173,174]. To compare, combusting 1 mg of hydrogen–oxygen mixture in the proportion of 2:1 gives 15.8 J of energy [125]. This means that, in practice, thermonuclear fuel will be about 200 million times more energy-dense than a chemical fuel, and in theory, it may be about 20 trillion times more energy-dense (if 100% of the ^2^H–^3^H fuel mass is burnt). However, when roughly estimated, the R&D costs associated with thermonuclear power are much higher than the R&D costs of all other ASEs combined [162]. 

In artificial nuclear fusion, the most convenient (low-temperature) reactions require the rarest, most difficult-to-obtain, and/or most costly components. For example, lithium-6 is rarely found on Earth (the main lithium isotope is lithium-7). Tritium must be synthesized in nuclear reactors. Helium-3 can only be excavated on the Moon or obtained using a nuclear reactor from tritium [162]. The fusion reactions with the most accessible components (e.g., hydrogen or helium-4) will be totally ineffective in small reactors [161]. This further increases the R&D and production costs of nuclear fusion power.

Furthermore, a thermonuclear reactor must contain a reproducing component that can meet the recurring demand for tritium. Tritium is environmentally dangerous due to its high radioactivity, and it is thus unfeasible to transport it from one place to the site of a power plant. Usually, a so-called breeding blanket is used, which will surround the camera trained on the nuclear fusion reaction and capture high-energy neutrons flying from it [168]. The blanket will contain melted lithium salts to ensure tritium synthesis through lithium nuclear fission [168]:
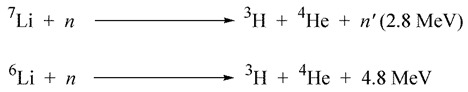
(13)

The cost of electricity produced in a thermonuclear power plant has not yet been evaluated with a sufficient degree of precision. The conversion of strong nuclear forces to electricity may be performed using turbines and a heat carrier fluid or via a direct energy conversion approach that transforms the kinetic energy of the particles into voltage [168]. The latter is currently deemed a better choice with respect to commercial feasibility.

## 5. Summary for ASE

In Table 1, we summarized the information about ASE in the context of the main research question.

## 6. Power Flows

To evaluate the potential of ASE within the “Spaceship Earth” paradigm means we have to assess the impact on the planet of not the very ASE but also

(1) its net use, i.e., its use on the scale of the Earth;
(2) the density and distribution of its use across the Earth’s surface;
(3) the harm that one ASE power plant causes or may cause.

Having assessed these factors, one can estimate the global effects of using an ASE on the planetary scale.

It is quite obvious that one windmill is harmless to the planet. Likewise, one solar power plant will not lead to negative effects, such as local climate changes, the migration of animals on the scale of a continent or mass extinctions. The local harm to the biosphere caused by a single geothermal station is in any case higher than that caused by a wind power plant or solar station, and the possible radioactive contamination resulting from a nuclear plant accident is many hundreds, even thousands, of times greater than that caused by a geothermal station.

**Figure 3:**
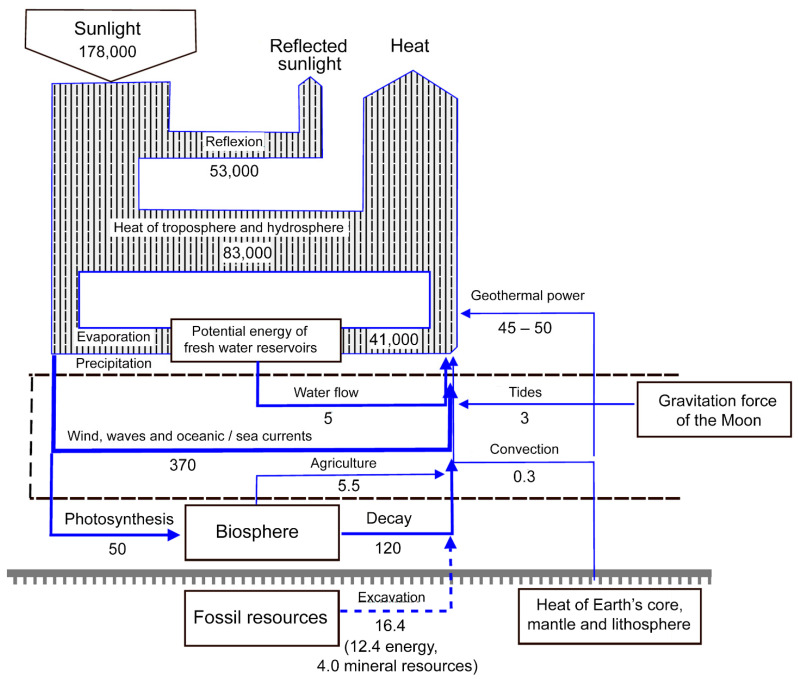
The most important power flows in nature and human activity, TW [4,19]. Adapted with permission from Ref. [19]. 1983, Wolfgang Sassin.

The Spaceship Earth paradigm considers the net energy flows that humanity will generate when using these ASEs, within a definite time scope. Figure 3 shows the power flows present in nature. It is useful for us to assess the degree of a given ASE’s anthropogenic influence on the planet. 

At present, humanity’s total annual power consumption is a little more than 20 TW [175]. We expect the world population to reach 15–20 billion people by 2100. We should also take into account the mean adjustment coefficient of 1.68, which stands for the ratio of the energy demand of the current generation to that of their parents [4] (much greater use of mobile devices, PCs, and video game devices; higher mobility, etc.). As a result, we may value the power demand of humanity in 2100 as approximately 100–130 TW. The 2–2.5-fold increase in population in the next three generations may lead to a 5–6.5 increase in power consumption.

We can clearly see that such power consumption, and the associated production of energy, are comparable with the power fluxes of several natural processes on the planetary scale (Figure 3). This means that our production and consumption of energy extracted from natural processes (ASE)—which is intended to reach 100%, or thereabouts—will be equal to, or at least of the same order of magnitude as, several of the Earth’s natural processes. This paradox is usually called “washing an elephant” [4,175]—we are trying to wash an elephant in a pond that is comparable to it in volume and seeking not to lose any water when the elephant is immersed in it. 

From Figure 3, it is clear that relying exclusively on one or even two ASEs will do no good for humans or the biosphere. 

For instance, supposing that the intergenerational increase in power consumption will soon be stopped or diminished (with a coefficient of around 1), and humanity will begin to consume “only” 40–50 TW by 2100. Theoretically, we may obtain all the energy we need from geothermal sources. However, this will destroy the biosphere and lithosphere in less than one generation.

Similarly, using only wind and solar power to generate all the energy humanity will need will surely lead to planetary disasters too, such as unexpected climate turbulence (with subsequent tornadoes, showers and droughts), changes in global wind currents and possible global haze or cloud cover events created by the removal of heat by solar power plants. These are very cautious suggestions of what may await humanity in the next century if all its energy demands are met using the two ASEs that are generally regarded as the most promising today (sun and wind).

Once again, relying merely on sea wave power and/or OTEC would cause changes in global sea currents, the destruction of the ocean biosphere, and the mass extinction of oceanic species. Furthermore, exclusively employing tidal power would slow down the rotational speed of the planet for several seconds, which would also cause natural disasters.

It may seem that 100–130 TW is a negligibly small figure in comparison with the 53,000 TW of the sun’s reflection power (Figure 3). However, one must not forget that the presence of 15–20 billion people will cause a substantial change in the reflection properties of the Earth’s surface. Even today, the presence of 8 billion people has changed the physical properties of the Earth’s surface dramatically, especially in highly populated regions with megacities and contiguous peri-urban areas (e.g., East China, Honshu, Taiwan, India’s coastline, Kibera, and the more southern regions of the East African coast, down to Durban) [176,177]. Therefore, Figure 3 provides the best estimates of sunlight reflection. The real value of natural sunlight power that is reflected may be substantially less due to the anthropogenic influence.

## 7. Source of Energy—Carrier Problem

As we mentioned in the Introduction, the Spaceship Earth perspective necessitates considering the so-called battery problem. This regards the irreducibility of the energy carrier and the source of energy to each other, i.e., the problem of the source of energy and the carrier of the energy being different.

The hydrocarbons we currently use are not merely a source of energy. They are simultaneously a carrier of this energy and sometimes high-density carriers. Compared to the list of other non-carbon ASEs, hydrogen only has similar properties, and to a limited extent [178]. Hydrogen may be burnt in car engines as an energy carrier, just like petrol, but it may not be as easily transported by pipelines as natural gas or crude oil. Other non-carbon ASEs cannot be used where and when we need them (that is, we cannot install a “mini nuclear fusion reactor” on board a transportation vehicle, even theoretically). Therefore, keeping and redistributing the produced energy is a task that accompanies the use of almost all ASEs.

To date, our industrial civilization has established only one effective means of keeping and transporting energy. This is electricity. ASE plants in constant operation, i.e., nuclear plants or hydropower plants, may be connected to the stationary systems of electricity delivery and redistribution, i.e., electrical current collectors, power lines, high-voltage substations, transformers, distributing panels and other constituents of national electrical networks. 

However, the majority of ASE plants work on an intermittent basis. They cannot be deployed where and when we prefer. Besides, they may not generate electrical power at the time we need it. Therefore, they require electric batteries for the storage of produced electricity, be these vehicle batteries, consumer electronics accumulators or large industrial power storage stations. This also applies to prospective thermonuclear plants, which will require relatively long periods of the shutdown to extract the useful components from the blanket covering the synthesis camera for their further use [161,162].

Regardless of the types of batteries used today (acid, nickel-cadmium, nickel-zinc, nickel-metallic-hydride, lithium-ion, or lithium-polymer), their production, operation and disposal are all environmentally unsafe. The risks associated with batteries should always be taken into account when evaluating the total cycle of an ASE.

One can estimate the increase in battery production by 2100. In 2021, ASEs accounted for 38% of global energy production [175]. 10% was accounted for by nuclear power, 15% by hydropower, 10% by solar and wind power together and the remaining 3% by biomass, hydrogen, geothermal and tidal power stations combined [175]. We must disregard the proportions contributed by nuclear power (10%) and hydropower (15%) production, as these do not require the use of industrial battery stations. This means that today, humanity produces industrial batteries to accommodate the 20 TW × 0.13 = 2.6 TW power flux from ASEs. In reality, more batteries are used, as we have neglected the batteries used in electric cars and consumer electronics batteries. We can therefore double this number, viz. 5.2 TW.

In the case of

(1) 100–130 TW power consumption in 2100;
(2) total zero-carbon energy (the proportion of ASE use is 100%);
(3) 50% presence of electric vehicles (with the remainder of vehicles powered by on-board hydrogen),

we can procure around 100–130 TW × 0.85 = 85–110 TW, which will require supplementary industrial and transport batteries. That is, as a result of the energy transition, the demand for batteries in 2100 may be roughly evaluated as 16 to 21 times higher than today’s demand. 

The problems of batteries are further boosted by the following difficulties in connecting ASEs to electrical networks:

(1) Most existing electrical networks are built as national projects. There are very few cross-border, large-scale electricity transmission projects, such as France–Italy or USA–Canada;
(2) Most hydrocarbon-fueled heat power plants are spread evenly across the globe and, consequently, are easy to connect to any electrical network. There is no difference where to combust natural gas or fuel oil.

However, most of the ASE power plants will be located in a limited number of regions around the world and distributed very unevenly for the reasons described in Chapters 3 and 4. This will also elevate the cross-border, and even cross-continental, demand for electricity produced by ASE-related power, which stationary electrical networks will hardly be able to meet.

Only the large-scale use of electrical batteries may be an option, at least from the modern perspective. 

Even today, producing electrical accumulators is a very risky and environmentally harmful process on the planetary scale. The enormous quantity of batteries required by 2100 will definitely require new technologies of battery production, as well as principles of operation and utilization cycles, even if the demand for batteries is five to ten, let alone twenty, times higher than it is today.

## 8. Limitations of the Review

1. Not all ASEs received equal focus in our review. Some were discussed more carefully, and others more cursorily;
2. We tried to ensure a more detailed consideration of ASEs that are usually deemed “more exotic”, and steered away from areas of mainstream research or media discourse. We confined ourselves to a briefer analysis of the more common ASEs, such as wind or solar energy, primarily focusing on their side effects and negative environmental impacts, as much has already been written about these ASEs;
3. We did not provide exhaustive information about any of the ASEs. The size of this review would not have allowed it. In addition to this, there are plenty of books concerning all of the ASEs. Our intention was to consider using a combination of multiple ASEs together as the only possible means of decarbonization and to ensure the great energy transition of the twenty-first century;
4. Our review included data and performed their analysis in a manner customary to environmentalist academia. The problem of the energy transition of the twenty-first century is much broader. It necessarily includes business entities, legal practitioners, politicians, policymakers, managers of different levels and government bodies. A truly comprehensive and systemic analysis of the ASEs must involve discussing the aspects of management, law, social attitudes and business planning. We have not considered these in our review. In our future work, we shall focus on some of these issues from the sociological viewpoint.

## 9. Conclusions

We have analyzed the most promising alternative sources of energy. In contrast to the majority of reviews and articles, we used the Spaceship Earth paradigm.

ASEs effectively meet the task of decarbonization and preventing further climatic changes. However, analyzing ASEs from the Spaceship Earth perspective leads us to the conclusion that there is no perfect ASE. No ASE is universally applicable, and no single one can be used everywhere on the planet with minimal expenses and maximal energy output.

Relying on a limited number of “preferred” ASEs (the preference may be dictated by political motives, the profit–loss rationale, or other business considerations) on the scale of the entire planet may be more dangerous than burning hydrocarbons. The greenhouse effect may be diminished, but the harm to the Earth may be more critical.

Unfortunately, today, the research, development and industrial uses of several ASEs, along with their corresponding financial considerations, are often accounted for by political considerations, social and politconomical fashion, media controversy or other factors unrelated to science. This is the case for many countries, political blocks and geographical regions, including both high-income countries and low-income ones. The Spaceship Earth paradigm stresses that an ASE’s being renewable does not automatically entail its safety and eco-friendliness on the planetary scale. A typical common view is the opposite: the “good” (metaphorically speaking, “green”) renewable energetics is being opposed to the “bad” (metaphorically “black”) use of heating oil or nuclear power plants. Given that this is the “commoner” view, it may be neglected and even ignored. However, if it acquires the traits of an ideology, it may become dangerous. The 2022 fiction comedy movie *Le visiteur du futur* (“The Visitor From the Future”) is a perfect allegory for this. The protagonist is “split” between two realities. His first “incarnation” is the director of the largest nuclear power plant on Earth, built in France. This future is a dark and hellish post-apocalyptic nightmare following the explosion of this station. His second ego is an engineer engaged in “green” energy. That reality is bright, jocund and happy. The chief part of the audience of this movie will stay unaware that an improper global use of a “green” ASE may cause much more harm to the planet than an explosion of the largest “black” nuclear plant.

In the nearest future (before 2030) there must be a triage procedure that would allocate financial, human and other resources among different ASEs. From the Spaceship Earth viewpoint, any ASE should be evaluated in the following terms:

(1) cost of construction of energy plants and infrastructure;
(2) operational costs;
(3) cost of the electricity generated;
(4) local and global environmental impact of the ASE itself;
(5) local and global environmental impact of the full cycle of the ASE;
(6) ability to be switched to national electrical networks;
(7) electrical battery/power stations requirements for energy transportation;
(8) energy production efficiency coefficient;
(9) energy output;
(10) geographical distribution of such power plants and the ability of the ASE to solve the energy inequality problem.

As one can anticipate, many of these parameters are mutually exclusive. Therefore, in the triage procedure for any ASE, the research teams, politicians and business representatives have to consider the given ASE as a compromise between these parameters.

Most importantly, the triage should not assess an ASE from a local viewpoint, be it the production of energy, consumption of energy or material chains necessary for using the ASE. Only the *global Spaceship Earth perspective*, where our planet is considered as a whole unit, may give an opportunity to estimate the environmental impact for the full cycle of an ASE, from the R&D stage to the utilization of waste the ASE will generate on the planetary scale.

From the Spaceship Earth perspective, we may conclude that using all ASEs in different proportions in various regions of the planet, where their harm to the planet and humanity can be minimized and, on the contrary, their efficiency maximized, would give humanity the opportunity to decarbonize the Earth and make the energy transition in the most effective way.

## Figures and Tables

**Table 1:** Pros and contras of different ASE in the context of their practicability/safety within the Spaceship Earth paradigm.

Source of Energy	Positive Features	Negative Features
Biofuels		
Solid	(1) simplicity of production, (2) ability to be a part of a closed resource cycle (within the borders of a self-sustained household), (3) broad availability of ingredients, (4) absence of special storage regime, (5) very long service lifetime, (6) inexpensiveness	(1) increase in carbonization, (2) deforestation, (3) practicability in limited regions of the Earth with forests
Liquid	(1) they are already widely used, (2) simplicity of equipment, (3) diversity of the fuels obtained	(1) relatively high cost of production, especially for biomethanol, (2) necessity in vast land or sea coast farming grounds, (3) large distortion of biosphere, (4) substantial increase in carbonization
Gaseous	(1) possibility to be received from waste (2) very low cost of production	(1) biomethane is a full analog of methane excavated from the Earth’s crust. Therefore, the carbonization is the same as for its industrial analog
Nuclear power	(1) large availability of the necessary elements (at least now), (2) simplicity of construction, (3) primitive operation cycle, (4) long service times, (5) it is already used for almost a century	(1) very high hazard of disaster events (meltdowns, leaks or explosions) with relatively small probability of these events, (2) necessity to dig exhausted radioactive fuel into the ground, (3) slow radioactive destruction of facilities, (4) vulnerability to human errors, (5) stimulation of energy inequality across the globe
Hydrogen	(1) eco-friendliness of the vehicles, (2) the safest fuel far to the environment known thus, (3) it may be stored in cylinders, absorption metal capacitors or generated on-board of vehicles	(1) perplexed colored classification, (2) complexity of production of “green” H_2_, (3) enormous production costs of “green” H_2_, (4) eco-hostility of production, hazardous technologies and reagents, involving alkalization of biosphere and pumping of gases to lithospheric splits, (5) “green” H_2_ is not a true source of energy; it is a means of re-pumping energy from one place to another on the planetary scale, (6) economical reasonability for production of mere “grey” H_2_, (7) large transportation costs, inability to build hydrogen pipelines due to physical properties of H_2_, (8) high explosiveness of its mixture with oxygen in 2:1 proportion
Hydropower	(1) very low production costs, (2) constant switching to national electrical networks, no battery stations are required, (3) little maintenance supervision is required, (4) long service times, (5) it is already sufficiently tested in our times	(1) severe destruction of biota that is observed in 100% cases, (2) change of river flow rate, increase in water temperature, (3) negative influence on lithosphere and soil, (4) indirect carbonization of atmosphere with natural gas synthesized during algae decay in shallow water reservoirs
Wind power	(1) it reduces carbonization significantly, (2) it is already sufficiently tested in our times	(1) perpetual high level of noise, (2) radio (UHF) waves interference, influence on mobile networks and TV, (3) ability to generate infrasound especially hazardous to water and soil biota, (4) creation of vast zones of human and biotic alienation, (5) changes of wind rose, (6) economic reasonability only on sea shores, (7) long periods of inactivity (no electricity generation). Therefore, it is difficult to switch wind power plants directly to national electrical networks. Battery stations are almost always required
Solar power	(1) easy installation and use on a little scale (households, small business, small agricultural farms), (2) practicability in almost any conditions whither sunlight reaches, independently of the temperature regime in an area (e.g., in mountains, under water, in cosmic space)	(1) complex maintenance systems are required in power plants of industrial size, (2) it can be used effectively only within 40° North to 40° South and a limited number of polar/circumpolar regions, (3) even greater zones of social and biotic alienation are being created than for wind power plants of comparable power output, (4) production cycle and installation of solar elements are dangerous to the environment, (5) ability to cause a drop in air temperatures in a local area and cloud formation that will decrease the efficiency of a solar power plant (self-suppression), (6) utilization of out-of-the-service power plants is environmentally dangerous, (7) long periods of inactivity (no electricity generation). Therefore, it is difficult to switch wind power plants directly to national electrical networks. Huge battery stations are always required
Geothermal power	(1) effectiveness in risky regions of elevated seismic and volcanic activity	(1) it is not widely used thus far, (2) the places where it can be used are spread very unevenly across the planetary surface, the regions of use are limited, (3) very high environmental impact, (4) high level of noise
Sea tidal power		(1) extensive use on the planetary scale will cause a decrease in the Earth’s rotation rate, (2) very high construction and maintenance costs
Ocean thermal power		(1) it is insufficiently researched thus far, (2) it may be effective only in equatorial and tropical zones of the World ocean, i.e., in the places where the temperature difference between water surface and deeper layers is maximal (around 25 °C)
Wind wave power	(1) it utilizes an unobvious but omnipresent source of energy	(1) it is insufficiently researched thus far, (2) cargo ships with batteries or constructing immense electrical networks on sea bottom are needed
Thermonuclear power	(1) it is almost a “perpetuum mobile” source, (2) it may cover energy demand of the whole humanity, (3) it exceeds any other ASE by efficiency coefficient and energy net value	(1) research and development (R&D) costs are tremendous; they exceed the total R&D costs for all other ASE, (2) it is not tested yet, either in laboratory or in industry, (3) risk of radioactive contamination is high, (4) hard-to-find or hard-to-synthesize substances are needed, (5) very short service life is foreseen due to high-energy neutron damage of facilities (less than a year), (6) electricity production costs are difficult to estimate

## Data Availability

Not applicable.

## Supplementary Materials

The following supporting information can be downloaded at: https://www.mdpi.com/article/10.3390/ijerph192316286/s1, Schematics. Classification of alternative sources of energy (ASEs).
