# Supplementary figures and images for: Using Alternative Sources of Energy for Decarbonization: A Piece of Cake, but How to Cook This Cake?
Source: Int J Environ Res Public Health. 2022 Dec 5;19(23):16286. doi: 10.3390/ijerph192316286 (PMC9735948; doi:10.3390/ijerph192316286)

**Schematics.** Classification of alternative sources of energy (ASEs).

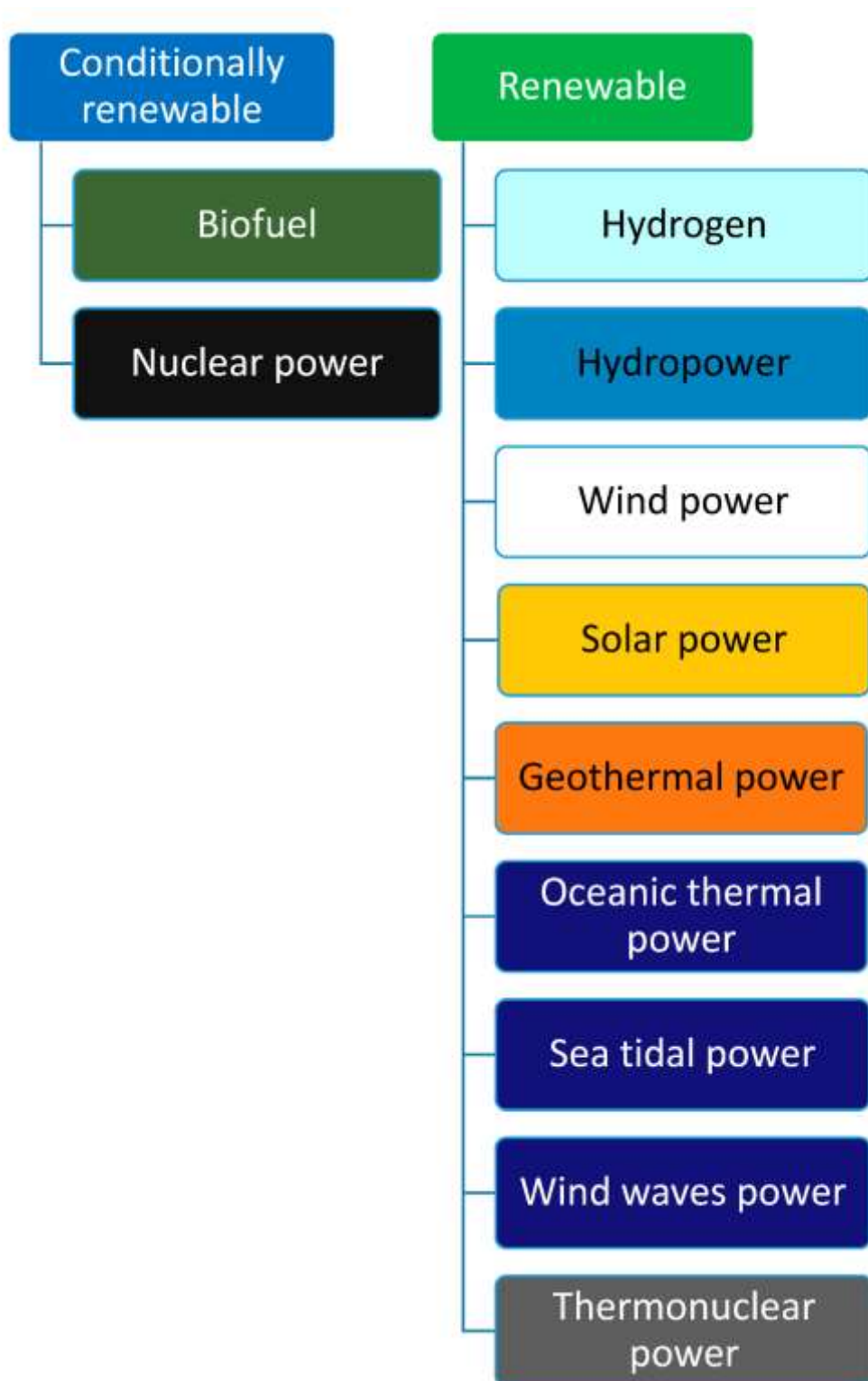

Supplement: Supplementary file 1 [file ijerph-19-16286-s001.zip › ijerph-2018831-supplementary.pdf]
